# Supplementary material for: Improved Delivery Performance of n-Butylidenephthalide-Polyethylene Glycol-Gold Nanoparticles Efficient for Enhanced Anti-Cancer Activity in Brain Tumor
Source: Cells. 2022 Jul 11;11(14):2172. doi: 10.3390/cells11142172 (PMC9325228; doi:10.3390/cells11142172)
Supplement: Supplementary file 1 [file cells-11-02172-s001.zip › cells-1753919-supplementary.pdf]

Improved Delivery Performance of n-butylidenephthalide-Polyethylene Glycol-Gold Nanoparticles Efficient for enhanced Anti-Cancer Activity in Brain Tumor

Supplementary data

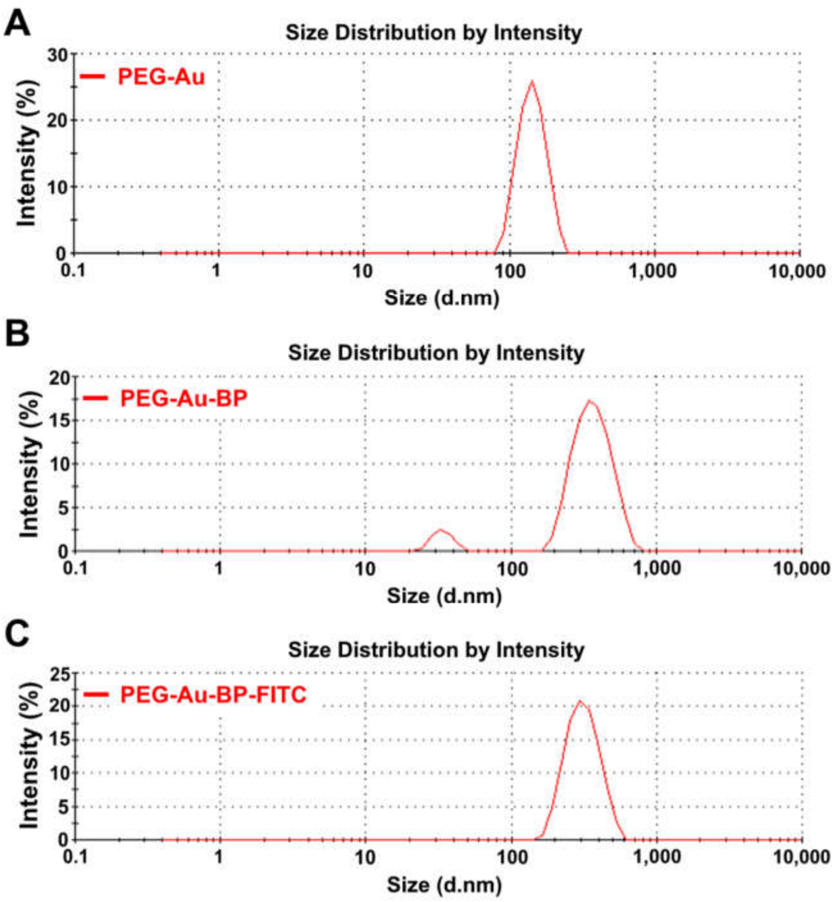

Figure S1. Size Distribution intensity measured by DLS assay. The as-prepared materials: (A) PEG-Au, (B) PEG-Au-BP and (C) PEG-Au-BP-FITC, were also subjected for DLS measurement. The diameter of each sample was quantified as 177.5±5.2, 246.27±7.0 and 288.2±4.2, repectively. Results are presented as one of three independent experiments.

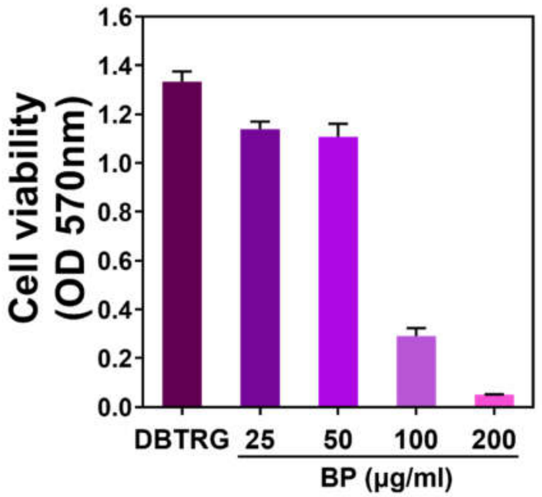

Figure S2. The concentration of IC_{50} for BP in the DBTRG brain cancer cell line. BP was prepared for variou s concentrations: 25, 50, 100, and 200 μg/mL. DBTRG cells were treated with various concentrations of BP. After 24 hours of incubation, the cell viability of DBTRG cells in each group was measured by MTT assay. After further calculation, the IC_{50} of BP in the DBTRG cell line was at the concentration of 50 μg/mL. Therefore, 50 μg/mL of BP was applied for the experiments.

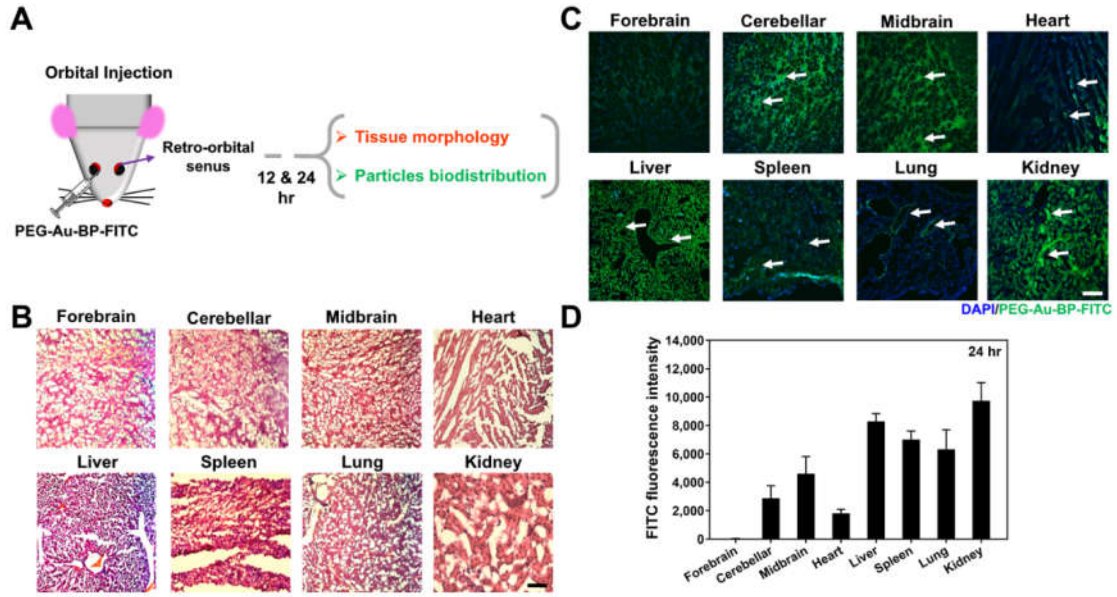

Figure S3. Tissue morphology and particle distribution after PEG-Au-BP treatment for 24 hours in an animal model. (**A**) The illustration indicated the mice were injected with PEG-Au-BP nanocarriers through retroorbital sinus injection. (**B**) Each tissue was stained with H&E staining to observe the tissue integrity after the treatment. The images indicated that the organs would not be harmed by PEG-Au-BP. (**C**) The histological images demonstrated the particle distribution in each organ. The white arrows presented for the FITC labeled PEG-Au-BP nanocarriers (green color). Cell nuclear were stained with DAPI solution (blue color). Scale bar equals to 50 um. (**D**) The FITC fluorescence intensity of PEG-Au-BP in each organ was quantified, where the nanocarriers could be observed in most organs/tissues. However, the quantified results at 24 hours were found to be higher in brain tissue when compared to the 12-hour injection, indicating the extended retention period in brain.

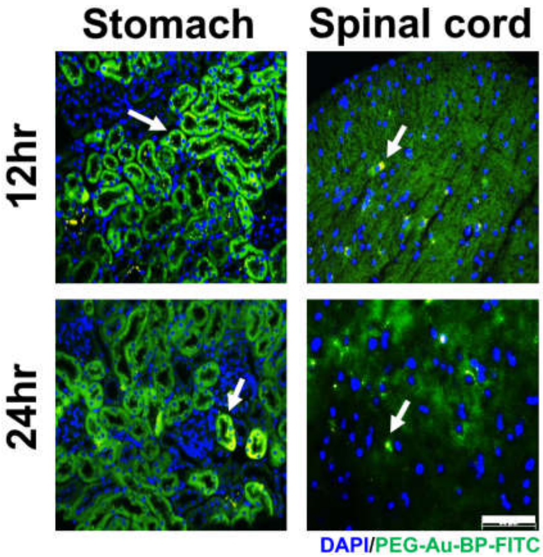

Figure S4. Particle distribution in stomach and spinal cord tissue after PEG-Au-BP treatment for 12 and 24 hours in an animal model. The histological images demonstrated the particle distribution in stomach and spinal cord tissue. The white arrows indicated the FITC labeled PEG-Au-BP nanoparticles (green color). Cell nuclear were stained with DAPI solution (blue color). Scale bar equals to 50 um.
